# Ultrasound guided endoscopic combined Intrarenal surgery – 10 steps for the success

**DOI:** 10.1590/S1677-5538.IBJU.2022.0029

**Published:** 2022-04-10

**Authors:** Fabio C. Vicentini, Kayann Kaled Reda El Hayek, Marcelo Szwarc, Rodrigo Perrella, Priscila Kuriki, David Cohen, Daniel Beltrame, Carlos Alfredo Batagello, Claudio Murta, Joaquim Francisco de Almeida Claro

**Affiliations:** 1 Hospital Brigadeiro - Centro de referência em Saúde do Homem São Paulo SP Brasil Hospital Brigadeiro - Centro de referência em Saúde do Homem - São Paulo, SP, Brasil

## Abstract

**Background:**

Endoscopic combined intrarenal surgery (ECIRS) has been used to treat complex kidney stones (1). The combined use of ultrasound (US) has the potential to improve safety and reduce radiation exposure, however, it is still underutilized (2).

**Objectives:**

Our objective is to describe, in a step-by-step manner, the ultrasound-guided ECIRS (USG ECIRS) technique, in order to facilitate learning by urologists.

**Materials and Methods:**

We describe the 10 standardized steps that we recommend to achieve a good outcome, based on our previous experience on a high-volume kidney stone center. We recorded a case of a 37-year-old female patient with complex bilateral kidney stones that underwent a left simultaneous combined retrograde and antegrade approach. The 10 described steps are: 1 - case evaluation with CT scan (3); 2 - preoperative care with antibiotics and tranexamic acid; 3 - warm-up and training with phantoms; 4 - patient positioning in Barts flank free position; 5 - retrograde nephroscopy with flexible ureteroscope; 6 - US and endoscopic guided puncture; 7 - tract dilation under endoscopic view; 8 - stone fragmentation; 9 - status free checking and 10, kidney drainage. Images were captured by external and internal cameras, promoting a complete understanding of the procedure. The patient has signed a written informed consent form.

**Results:**

Puncture was achieved under US guidance with one attempt. Another puncture was necessary in the lower pole, parallel to the initial puncture, due to a large fragment. Surgical time was 140 min. Stone-free status was verified by retrograde and antegrade view. Kidney drainage was done with ureteral stent on string, removed after 7 days. Hb drop was 1.1 Hb/dL. The first postoperative day CT scan showed no residual stones and no complications. The patient was discharged after the CT and urethral catheter removal.

**Conclusion:**

The USG ECIRS seems to be a very efficient and reproducible technique for the treatment of complex kidney stones. Its use should be widespread.
